# Predictors of Poor Pneumonia Outcomes in Older Adults: A Multicentered Follow‐Up Study

**DOI:** 10.1002/hsr2.70666

**Published:** 2025-04-18

**Authors:** Tegene Atamenta Kitaw, Addisu Getie, Melesse Abiye Munie, Yabibal Asfaw Derso, Solomon Gebremichael Asgedom, Ribka Nigatu Haile

**Affiliations:** ^1^ Department of Nursing, College of Health Science Woldia University Woldia Ethiopia; ^2^ Department of Nursing, College of Medicine and Health Sciences Debre Markos University Debre Markos Ethiopia; ^3^ Department of Surgical Nursing School of Nursing, College of Health Sciences and Comprehensive Specialized Hospital, Aksum University Axum Ethiopia

**Keywords:** poor outcome, predictors, severe pneumonia

## Abstract

**Background and Aims:**

Severe pneumonia is a leading cause of mortality among older adults, particularly in low‐income countries like Ethiopia. Despite treatment advancements, the role of different factors in predicting poor outcomes remains understudied. This study aims to assess early indicators poor outcomes in severe pneumonia among older adults.

**Methods:**

A multicenter retrospective cohort study was conducted involving 422 older adults aged over 65 years. Kaplan–Meier survival curves and log‐rank tests were utilized to compare survival across categories. Cox regression analysis was performed to evaluate the association between these factors and poor outcomes, while adjusting for potential confounders.

**Results:**

Of the patients, 13.27% (95% CI: 10.18, 16.88) died from severe pneumonia. CHF (AHR = 2.35, 95% CI: 1.23, 4.49), DM (AHR = 2.52, 95% CI: 1.23, 5.14), and COPD (AHR = 2.59, 95% CI: 1.39, 4.81), elevated creatinine (AHR = 1.63, 95% CI: 1.63, 2.69), and low platelet count (AHR = 2.43, 95% CI: 1.78, 5.34) were significantly associated with poor outcomes.

**Conclusion:**

Thrombocytopenia and high creatinine levels may serve as early indicators of poor outcomes in severe pneumonia among older adults. Monitoring these markers is critical for guiding interventions and improving patient outcomes.

## 1 Introduction

Pneumonia refers to an infection of the lungs or pulmonary parenchyma. Community‐acquired pneumonia (CAP) is a subtype of pneumonia characterized by an acute infection of the pulmonary parenchyma acquired outside of a healthcare setting. It is typically diagnosed within 48 h of hospital admission, distinguishing it from hospital‐acquired pneumonia (HAP) [1]. Severe CAP is a severe disease with poor outcomes and requiring a higher level of care [2]. Severe CAP is defined as the presence of severe acute respiratory failure (ARF) needing invasive mechanical ventilation (IMV) and/or septic shock with organ system dysfunction [3].

Severe CAP is one of the most common infectious diseases and is a significant cause of mortality and morbidity in older people [4]. CAP is a serious health concern, with substantial ramifications for healthcare systems globally. Despite substantial breakthroughs in prevention via immunizations, new quick diagnostic tests, and medicines, CAP management still has significant drawbacks [5]. Malnutrition, household crowding, and exposure to indoor air pollution from domestic combustion of biomass fuels may also contribute to the burden of pneumonia in low‐income countries [6].

Despite advances in modern medicine, CAP continues to be a potentially fatal disease. Approximately 20% of the patients with CAP require hospital admission. One‐fourth of patients with severe CAP require IMV [7]. According to the World Health Organization (WHO) statistics, lower respiratory tract infections (LRTIs) are the major infective cause of mortality worldwide accounting for 6.1% of mortality [8]. LRTI accounted for 2.8 million deaths and the loss of 115 million disability‐adjusted life years [9].

Emerging evidence has highlighted the utility of markers such as elevated blood urea nitrogen (BUN) [10], serum creatinine, low platelet count [11], abnormal white blood cell (WBC) count [12], elevated C‐reactive protein (CRP) [13], low hemoglobin, and high lactate dehydrogenase (LDH) [14] levels as predictors of adverse outcomes. In patients with pneumonia, thrombocytopenia serves as a notable indicator of poor prognosis, as it is associated with severe intravascular coagulation and the development of severe sepsis [15].

In low‐income countries, mortality related with CAP tends to be higher, as proven report of mortality rate of 23% in Cambodia, 19% in Senegal, 18% in Uganda, 16% in Central African Republic, and 14.6% in Malawi [16]. In sub‐Saharan Africa, estimates suggest 4 million episodes of pneumonia each year, resulting in more than 200,000 deaths [17]. In Ethiopia, the mortality rate of CAP among admitted patients tends to 20.2%. Patients older than 65 years, respiratory rate > 30 breaths/min, and comorbid tuberculosis were associated with poor treatment outcomes. The mean duration of hospital stay following infected with CAP is 11.49 days [18].

Despite the significant burden of severe pneumonia on older adults, research in developing nations like Ethiopia has predominantly concentrated on pediatric cases, leaving older adults underrepresented. There is a lack of studies investigating the unique challenges and predictors of poor pneumonia outcomes in this population. Additionally, clinical guidelines often lack specific recommendations tailored to older adults, making it difficult to identify and manage those at heightened risk effectively. This study underscores the critical need to explore early indicators of poor pneumonia outcomes in older adults. By addressing this gap, we aim to enhance understanding and provide insights that can guide healthcare professionals in implementing timely and effective interventions, ultimately improving recovery rates and reducing mortality in this vulnerable age group.

## 2 Methods

### 2.1 Study Setting, Study Period, and Study Design

This research took place in hospitals across North Wollo Zone, located in Ethiopia's Amhara region. The administrative center, Woldia, is positioned 521 km from Addis Ababa and 360 km from Bahir Dar. A retrospective cohort study was carried out between August and September 2023. Three hospitals—Woldia Comprehensive Specialized Hospital, Mekiet Primary Hospital, and Wadila Primary Hospital—were randomly chosen from a total of five facilities. These facilities serve a diverse population and offer a wide range of healthcare services, including comprehensive inpatient and outpatient care for both acute and chronic illnesses. Each hospital is equipped with 24‐h emergency departments to handle urgent medical cases, along with maternal and child health services that encompass antenatal, delivery, and postnatal care. They also provide specialized treatments for severe conditions like pneumonia, featuring intensive care units and respiratory support. Additionally, these hospitals engage in preventive care and health education initiatives to promote overall community health. A multicentered retrospective cohort study was conducted from August 1 to September 30, 2023.

### 2.2 Population, Eligibility, and Sampling Technique

The source population was all older adults (≥ 65) with severe CAP who were admitted at North Wello hospitals. Whereas the study population consists of patients admitted to selected hospitals (three hospitals). All medical records of older adults (≥ 65) with severe CAP in selected North Wello hospital during the defined follow‐up period (from January 1, 2020 to December 31, 2021) were eligible to be included in the study. From five hospitals, three hospitals (Woldia Comprehensive Specialized Hospital, Mekiet Primary Hospital, and Wadila Primary Hospital) were selected by simple random sampling. A 2‐year estimated number of older patients flow with severe CAP was obtained from each selected hospital. The total number of patients admitted during the defined period was 417 at Woldia Comprehensive Specialized Hospital, 224 at Mekiet Primary Hospital, and 204 at Wadila Primary Hospital, resulting in 845 patients. Proportional allocation was performed by dividing the total by the sample size (845/422), yielding a factor of nearly 2. This provided sample sizes of 208 from Woldia, 112 from Mekiet, and 102 from Wadila, totaling 422 patients (208 + 112 + 102 = 422).

### 2.3 Variables and Definitions

The dependent variable is a poor outcome, which refers to the occurrence of death in patients with severe pneumonia during the follow‐up period. This study considered different explanatory variables under sociodemographic, clinical characteristics on admission, comorbidities, treatment‐related factors, and laboratory markers. In this study, event of interest referred to death from severe CAP during the follow‐up time. Censored refers to patients who were not observed to experience the outcome of interest during the follow‐up period, including those who recovered, were lost to follow‐up, or were transferred to different healthcare facilities. Incomplete patient chart represents charts that have no date of admission, discharge, and final outcome. We have clarified the definition of severe CAP. A CURB‐65 score of 2 or higher indicates severe pneumonia, while patients classified as IV or V on the Pneumonia Severity Index (PSI) are also categorized as having severe pneumonia [19].

### 2.4 Data Collection Tools

Structured checklist was used to collect the data. The checklist contains five main sections; Part I aimed at collecting information on basic sociodemographic variables of the patients, Part II consisted of questions required to gather information on patient comorbidities status, Part III includes questions to assess clinical characteristics of admission, Part IV deals about laboratory findings, and Part V contain question regarding treatment‐related factors.

### 2.5 Data Processing and Analysis

The data were coded, cleaned, and entered using Epi‐Data version 4.2. Analysis was conducted using STATA Version 17.0. Descriptive statistics, including measures of central tendency and dispersion for continuous data, and frequency distributions for categorical data, were performed. Kaplan–Meier curves were employed to visualize survival trends, while log‐rank tests were used to evaluate group differences. Multicollinearity among predictors was assessed through variance inflation factors (VIF) before running a Cox proportional hazards regression model, which examined associations between independent variables and pneumonia outcomes. Schoenfeld residuals validated the proportional hazards assumption, and model accuracy was confirmed using a Cox–Snell residual plot. Variables with a *p* value of ≤ 0.25 in the bivariate analysis were included in the multivariable model, and those with a *p* value of ≤ 0.05 were considered statistically significant. Hazard ratios (HRs) with 95% confidence intervals and *p* values were used to evaluate the strength of associations. The findings were presented using text, tables, and graphs.

### 2.6 Ethical Approval Details and Informed Consent

All data were fully anonymized before being accessed. The study was reviewed and granted a waiver for the requirement of informed consent by the Woldia University Institutional Review Board (Protocol Number: WDU/IRB001). Since this study utilized retrospective data, direct consent from participants was not applicable. However, the respective hospitals waived the consent requirement after reviewing the proposal for ethical considerations. Information obtained from the records was kept anonymous to ensure confidentiality. The authors had no access to information that could identify individual participants during or after data collection.

## 3 Results

### 3.1 Sociodemographic and Clinical Characteristics of the Participants

The median age of the participants was 70 years (IQR = 68–74 years). The majority of participants (64.7%) were male. Concerning clinical characteristics, the vast majority of patients experienced cough (96.0%) and shortness of breath (79.9%). Additionally, 46.9% of the participants reported chest pain. The *χ* ^2^ test showed that age over 75 years (*p* = 0.015), male sex (*p* = 0.031), the presence of chest pain (*p* = 0.024), and oxygen saturation levels below 90% (*p* = 0.010) were significantly associated with higher mortality from severe pneumonia (Table 1).

**Table 1:** Sociodemographic and clinical characteristics of older adults with severe community acquired pneumonia.

Variable	Category	Censored (%)	Death (%)	Total (%)	*p* value
Age in years	65–70	190 (51.9%)	19 (33.9%)	209 (49.5%)	0.015*
	71–75	122 (33.3%)	23 (41.1%)	145 (34.4%)	
	> 75	54 (14.8%)	14 (25.0%)	68 (16.1%)	
Sex	Male	232 (63.8%)	41 (73.2%)	273 (64.7%)	0.031*
	Female	134 (36.2%)	15 (26.8%)	149 (35.3%)	
Cough	No	14 (3.8%)	3 (5.4%)	17 (4.0%)	0.193
	Yes	352 (96.2%)	53 (94.6%)	405 (96.0%)	
Fever	No	83 (22.8%)	6 (10.7%)	89 (21.1%)	0.097
	Yes	283 (77.2%)	50 (89.3%)	333 (78.9%)	
Shortness of breathing	No	77 (21.0%)	8 (14.3%)	85 (20.1%)	0.241
	Yes	289 (79.0%)	48 (85.7%)	337 (79.9%)	
Chest pain	No	204 (55.8%)	20 (35.7%)	224 (53.1%)	0.024*
	Yes	162 (44.2%)	36 (64.3%)	198 (46.9%)	
Fatigue	No	58 (15.8%)	17 (30.4%)	75 (17.8%)	0.121
	Yes	308 (84.2%)	39 (69.6%)	347 (82.2%)	
Headache	No	117 (31.9%)	23 (41.1%)	140 (33.2%)	0.058
	Yes	249 (68.1%)	33 (58.9%)	282 (66.8%)	
Vomiting	No	185 (50.5%)	36 (64.3%)	221 (52.4%)	0.076
	Yes	181 (49.5%)	20 (35.7%)	201 (47.6%)	
Loss of appetite	No	358 (98.1%)	55 (98.2%)	413 (97.9%)	0.089
	Yes	8 (1.9%)	1 (1.8%)	9 (2.1%)	
Arthralgia	No	134 (36.5%)	29 (51.8%)	163 (38.6%)	0.162
	Yes	232 (63.5%)	27 (48.2%)	259 (61.4%)	
Oxygen saturation	< 90%	29 (7.9%)	19 (33.9%)	48 (11.4%)	0.010*
	90%–94%	260 (70.6%)	30 (53.6%)	290 (68.7%)	
	≥ 95%	77 (21.0%)	7 (12.5%)	84 (19.9%)	

* Represent significance levels at *p*‐values 0.05.

### 3.2 Comorbidities

A considerable proportion of participants (49.3%) had at least one underlying medical condition, with hypertension (29.4%) being the most common, followed by diabetes mellitus (DM; 15.2%) and congestive heart failure (12.6%). The analysis revealed significant differences in patient outcomes based on several variables. Comorbidity (*p* = 0.000), congestive heart failure (*p* = 0.001), DM (*p* = 0.000), hypertension (*p* = 0.017), and chronic obstructive pulmonary disease (COPD) (*p* = 0.000) were all significantly associated with higher death rates (Table 2).

**Table 2:** Comorbidity distributions of older adults with severe community acquired pneumonia.

Variable	Category	Censored (%)	Death (%)	Total (%)	*p* value
Comorbidity	No	207 (57.7%)	7 (12.4%)	214 (50.7%)	0.000
	Yes	159 (42.3%)	49 (87.6%)	208 (49.3%)	
Congestive heart failure (CHF)	No	328 (89.2%)	41 (73.2%)	369 (87.4%)	0.001
	Yes	38 (10.8%)	15 (26.8%)	53 (12.6%)	
Diabetes mellitus (DM)	No	327 (89.1%)	31 (55.4%)	358 (84.8%)	0.000
	Yes	39 (10.9%)	25 (44.6%)	64 (15.2%)	
Hypertension (HTN)	No	266 (72.9%)	32 (57.1%)	298 (70.6%)	0.017
	Yes	100 (27.1%)	24 (42.9%)	124 (29.4%)	
Chronic obstructive pulmonary disease (COPD)	No	299 (80.5%)	27 (47.8%)	326 (77.3%)	0.000
	Yes	67 (19.5%)	29 (52.2%)	96 (22.7%)	
Asthma	No	353 (97.2%)	54 (96.4%)	407 (96.4%)	0.994
	Yes	13 (2.8%)	2 (3.6%)	15 (3.6%)	
HIV/AIDS	No	357 (97.1%)	55 (98.2%)	412 (97.6%)	0.758
	Yes	9 (2.9%)	1 (1.8%)	10 (2.4%)	
Chronic liver disease (CLD)	No	357 (97.1%)	53 (94.6%)	410 (97.2%)	0.236
	Yes	9 (2.5%)	3 (5.4%)	12 (2.8%)	
Stroke	No	350 (95.2%)	52 (92.9%)	402 (95.3%)	0.363
	Yes	16 (4.8%)	4 (7.1%)	20 (4.7%)	
Chronic kidney disease (CKD)	No	345 (94.0%)	53 (94.6%)	398 (94.3%)	0.604
	Yes	21 (6.0%)	3 (5.4%)	24 (5.7%)	

### 3.3 Laboratory and Treatment‐Related Characteristics

Analysis of basic laboratory markers revealed that 24.6% of the total patient population exhibited leukocytosis. Furthermore, thrombocytopenia was observed in 19.4% of the study cohort. Regarding treatment‐related characteristics, it was noted that ~22.5% of the study sample required mechanical ventilation as part of their therapeutic management. The *χ* ^2^ tests revealed significant associations between several clinical variables and patient outcomes (censored vs. death). Specifically, WBC count (*p* = 0.000), platelet count (*p* = 0.000), hematocrit levels (*p* = 0.001), creatinine levels (*p* = 0.000), BUN (*p* = 0.002), and mechanical ventilation (*p* = 0.000) all showed significant differences in outcome distributions. Hemoglobin levels also had a significant association (*p* = 0.037), while random blood sugar did not (*p* = 0.347) (Table 3).

**Table 3:** Laboratory and treatment‐related characteristics of older adults with severe community acquired pneumonia.

Variable	Category	Censored (%)	Death (%)	Total (%)	*p* value
White blood cell (WBC)	< 4.5 × 10^9^/L	27 (6.4%)	0 (0.0%)	27 (6.4%)	0.000
	4.5–11 × 10^9^/L	271 (64.2%)	14 (3.3%)	285 (67.5%)	
	> 11.0 × 10^9^/L	68 (16.1%)	42 (9.0%)	110 (24.6%)	
Platelet count	< 150,000/µL	61 (16.7%)	42 (75.0%)	103 (24.4%)	0.000
	≥ 150 cells/L	305 (83.3%)	14 (25.0%)	319 (75.5%)	
Hemoglobin (HgB)	≤ 14.2 g/dL	174 (47.5%)	35 (62.5%)	209 (49.5%)	0.037
	> 14.2 g/dL	192 (52.5%)	21 (37.5%)	213 (50.5%)	
Random blood sugar (RBS)	≤ 39.8%	201 (72.3%)	24 (64.9%)	225 (71.4%)	0.347
	> 39.8%	77 (27.7%)	13 (35.1%)	90 (28.6%)	
Hematocrit (HcT)	≤ 116.3 mg/dL	143 (39.1%)	35 (62.5%)	178 (42.2%)	0.001
	> 116.3 mg/dL	223 (60.9%)	21 (37.5%)	244 (57.8%)	
Creatinine	< 0.5 mg/dL	16 (4.7%)	7 (12.7%)	23 (5.8%)	0.000
	0.5–1.5 mg/dL	300 (82.0%)	17 (30.9%)	317 (83.8%)	
	> 1.5 mg/dL	25 (7.3%)	31 (56.4%)	56 (10.4%)	
Blood urea nitrogen (BUN)	< 10 mg/dL	38 (10.4%)	5 (8.9%)	43 (10.2%)	0.002
	10–20 mg/dL	196 (53.6%)	17 (30.4%)	213 (50.5%)	
	> 20 mg/dL	132 (36.1%)	34 (60.7%)	166 (39.3%)	
Mechanical ventilation	No	298 (81.4%)	29 (51.8%)	327 (77.5%)	0.000
	Yes	68 (18.6%)	27 (48.2%)	95 (22.5%)	

Abbreviations: /L, per liter; µL, microliter; cells/L, cells per liter; g/dL, grams per deciliter; mg/dL, milligrams per deciliter.

### 3.4 Survival Status of Older Adults With Severe Pneumonia

In this study, throughout the follow‐up period, 13.27% (95% CI: 10.18, 16.88) of patients died from severe CAP. The overall median survival time to death from SCAP was 17 days with interquartile range (IQR) = 15–21. The total follow‐up time contributed by all study participants was 4596 ‐person day observation. The survival probability of time to death at 10, 15, and 20 days was 97.06%, 87.51%, and 52.50%, respectively.

### 3.5 Comparisons of Survival Functions of Different Categorical Variables

Kaplan–Meier survival curve and log‐rank test were computed to compare and estimate the survivor function among different groups of variables. In the Kaplan–Meier survival curve, one survivorship function curve located under another means the lower curve group has a lower survival status than the upper curve group or has a less desirable survival probability than the upper curve. Furthermore, the difference was described statistically by the log‐rank test.

**Figure 1:**
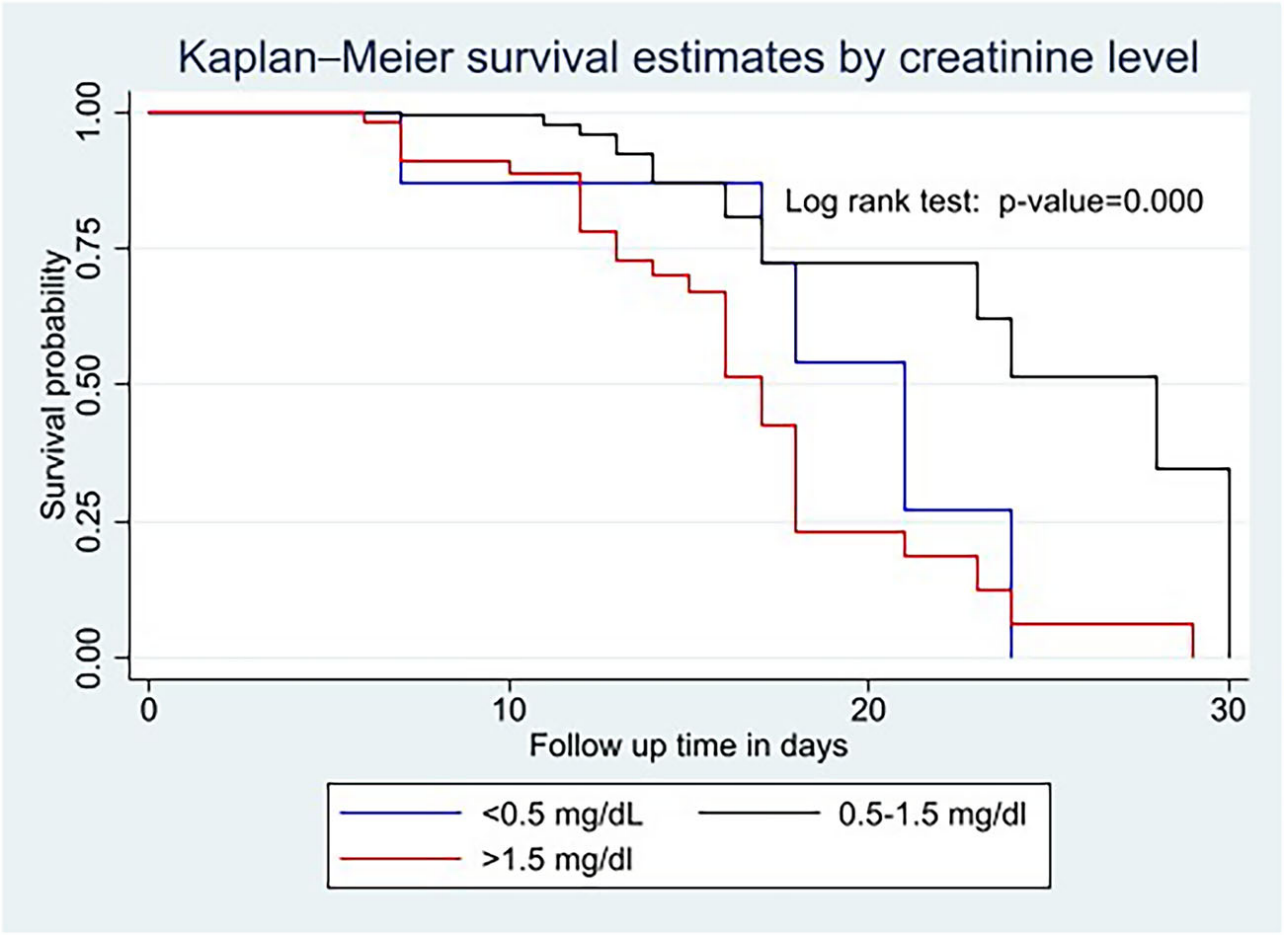
Kaplan–Meier survival curves and log‐rank tests by platelet count among older adults with severe pneumonia.

**Figure 2:**
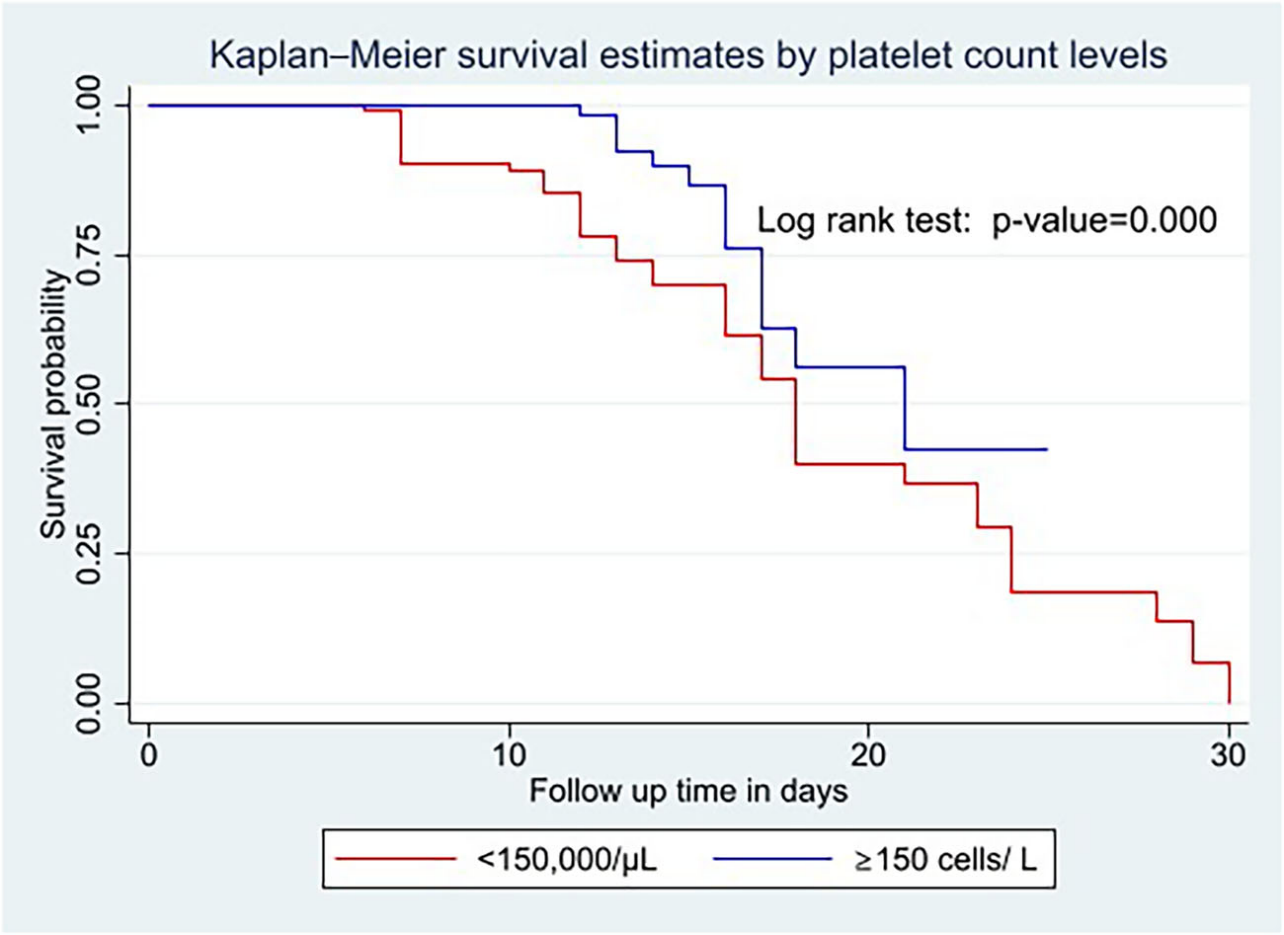
Kaplan–Meier survival curves and log‐rank tests by creatinine level among older adults with severe pneumonia.

Generally, the Kaplan–Meier survival curve and log‐rank test shows that older adults with platelet count bellow < 150,000/µL (Figure 1) and creatinine level of above 1.5 mg/dL (Figure 2) die within a short period of time than the comparison group.

### 3.6 Proportional Hazard Assumption Test

Proportional hazard assumption test by Schoenfeld residual revealed that the *ρ* statistic *p* value of all covariates is above 0.05 and global test *p* value is 0.1379 (> 0.05), which means that the Cox‐proportional hazard assumption is satisfied (Supporting Information S1: Table 1).

### 3.7 Goodness of Fit of the Cox Regression Model

**Figure 3:**
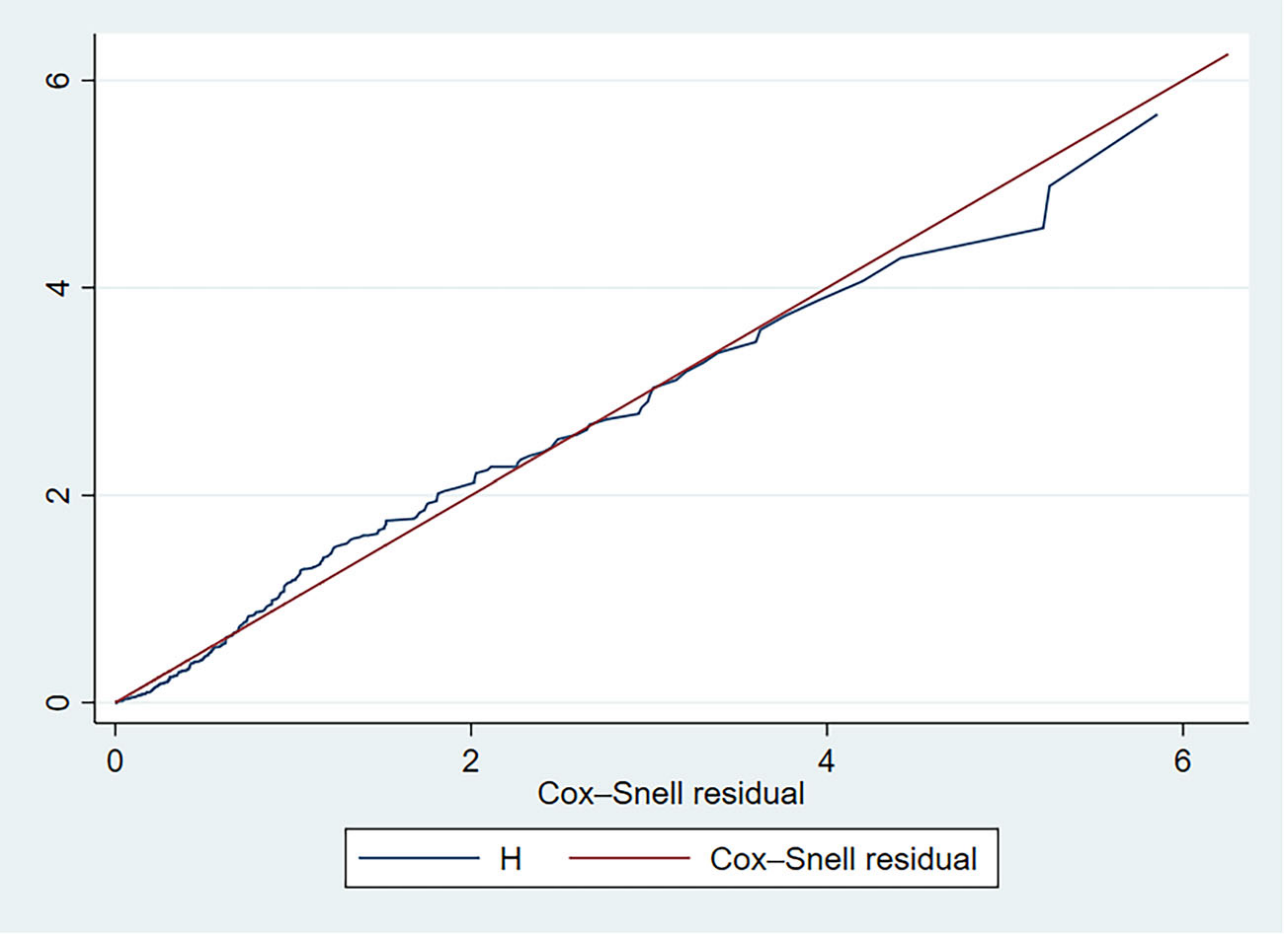
Cox–Snell residual plot to test the goodness of fit of the Cox regression model.

The goodness of fit for the Cox regression model was assessed using a Cox–Snell residual plot. Analysis of the plot revealed that the residuals align closely with the reference line (within a 45° alignment), indicating a strong fit between the model and the data (Figure 3).

### 3.8 Multicollinearity Test

VIF and tolerance values were used to check the existence of multicollinearity between variables. A VIF above 4 or tolerance below 0.25 indicated that multicollinearity might exist. In this study, the maximum VIF was 3.33 with a mean VIF of 1.52, and the minimum tolerance value was 0.3. Thus, there is no multicollinearity between covariates (Supporting Information S2: Table 1).

### 3.9 Adjusted Multivariable Analysis

After adjusting for age, sex, comorbidity, WBC level, and number of comorbidities, we found that creatinine, congestive heart failure, diabetes mellitus, COPD, and platelet levels were linked with poor outcomes. Individuals with CHF, DM, and COPD had significantly higher hazards of poor outcomes compared to the reverse group, with adjusted hazard ratios (AHR) of 2.35 (95% CI: 1.23, 4.49), 2.52 (95% CI: 1.23, 5.14), and 2.59 (95% CI: 1.39, 4.81), respectively. The hazard of mortality was 2.43 times higher among patients with a platelet count below 150,000/µL than the reverse group (AHR = 2.43, 95% CI: 1.78, 5.34). Furthermore, patients presenting with a creatinine level above 1.5 mg/dL have a 1.63 times higher hazard of mortality compared to patients with normal ranges of creatinine levels (AHR = 1.63, 95% CI: 1.63, 2.69). There is no association between BUN levels and poor outcomes of severe pneumonia (AHR = 1.16, 95% CI: 0.54, 2.09) (Table 4).

**Table 4:** Cox regression analysis to determine the relationship platelet count, blood urea nitrogen, and creatinine level with poor outcome of severe pneumonia.

Variables	Categories	CHR (95 CI)	AHR (95 CI)	*p* value
Age	65–70	1	1	
	71–75	1.02 (0.49, 2.54)	1.32 (0.81, 1.73)	0.245
	> 75	4.08 (2.85, 8.52)	2.81 (1.27, 6.19)	0.011
Sex	Male	1	1	0.230
	Female	0.89 (0.51, 1.56)	0.67 (0.34, 1.31)	
	Yes	3.33 (1.42, 7.78)	2.54 (0.85, 7.59)	
	Yes	5.46 (2.68, 11.10)	5.42 (2.13, 8.65)	
Congestive heart failure	No	1	1	0.010*
	Yes	4.91 (2.92, 6.24)	2.35 (1.23, 4.49)	
Diabetes mellitus	No	1	1	0.011*
	Yes	5.03 (3.01, 8.41)	2.52 (1.23, 5.14)	
COPD	No	1	1	0.003**
	Yes	3.91 (1.93, 7.24)	2.59 (1.39, 4.81)	
WBC	< 4.5 × 10^9^/L	2.34 (0.79, 4.98)	1.54 (0.85, 2.65)	0.091
	4.5–11 × 10^9^/L	1	1	
	> 11.0 × 10^9^/L	5.47 (3.08, 6.71)	2.19 (1.42, 4.46)	0.030*
Creatinine level	< 0.5 mg/dL	1.67 (0.65, 4.26)	1.21 (0.42, 3.12)	0.675
	0.5–1.5 mg/dL	1	1	
	> 1.5 mg/dL	3.59 (1.93, 6.68)	1.63 (1.66, 2.69)	0.017*
Platelet count	≥ 150 cells/L	1	1	
	< 150,000/µL	3.04 (1.59, 5.82)	2.43 (1.78, 5.34)	0.027*
Blood urea nitrogen	< 10 mg/dL	1.19 (0.43, 3.26)	0.45 (0.13, 1.53)	0.134
	10–20 mg/dL	1	1	
	> 20 mg/dL	1.57 (0.86, 2.87)	1.16 (0.54, 2.09)	0.547

*Note:* * and ** indicate statistical significance at *p* values of 0.05 and 0.01, respectively.

## 4 Discussion

In this study, over the follow‐up period, 13.27% of patients died from severe CAP. We found that CHF, DM, COPD, creatinine, and platelet levels were significant determinants of poor outcomes.

This study found 13.27% of patients die from severe CAP. This findings show higher mortality rates than those reported in studies conducted in Ethiopia (12.7%) [20] in 2023, Japan (6.7%) in 2017 [21], and Canada (6.83%) in 2010 [22]. However, our findings are lower than another studies conducted previously in Ethiopia in 2021, where the mortality rate was reported to be 24.27% in 2021 [23], and 20.2% in 2014 [18]. Furthermore, it was also found to be lower that reported in Thailand (45%) in 2021 [24] and Germany (23.1%) in 2015 [25]. The variation in mortality rates observed across different studies can be attributed to several factors, including differences in study populations, healthcare infrastructure, methodological approaches, treatment practices, and contextual factors. Variations in patient demographics, illness severity, and comorbidities may influence SCAP outcomes, along with disparities in healthcare resources and access to care. Methodological differences in study design and data collection methods may also contribute to discrepancies in findings. Additionally, variations in healthcare practices, treatment protocols, and cultural beliefs can impact mortality rates. It is also important to note that treatment protocols continue to evolve, which may contribute to differences in reported mortality rates over time.

Individuals with chronic heart failure (CHF), DM, and COPD face significantly higher risks of poor outcomes due to the combined effects of these conditions. This is also evident in other studies [26, 27]. CHF leads to impaired heart function, resulting in poor blood circulation and multiorgan dysfunction. DM causes systemic vascular damage and impaired immune response, increasing susceptibility to infections and complications. COPD, characterized by chronic lung inflammation and restricted airflow, exacerbates cardiovascular strain and increases the risk of respiratory failure. The overlap of these conditions creates a complex, interdependent health situation, with systemic inflammation, frequent hospitalizations, and treatment interactions complicating disease management. Together, these factors elevate the risk of adverse outcomes, such as disability, hospitalization, and premature death, highlighting the need for integrated care approaches to manage these comorbidities effectively.

Elderly individuals experiencing elevated creatinine levels were found to have a 1.63 times higher hazard of mortality from severe pneumonia when compared to patients within the normal range. Similarly, previous studies also revealed similar findings [23, 28]. This might explain by the fact that severe pneumonia often leads to dehydration due to high fever, increased respiratory rate, and decreased fluid intake, causing a reduction in kidney function and impaired creatinine clearance [29, 30]. Clinically, this suggests the importance of monitoring creatinine levels in elderly patients with SCAP, as it may help healthcare providers anticipate a poor outcome and plan appropriate management strategies. Additionally, identifying elevated creatinine levels early in the course of SCAP could prompt closer monitoring and more aggressive interventions to prevent further complications and improve outcomes in this vulnerable patient population. Implementing kidney care bundles has been shown to lower complications in patients with elevated creatinine. These bundles typically include careful monitoring of fluid balance, early identification of renal dysfunction, and timely interventions such as controlled hydration and medications to support kidney function, thereby preventing mortality associated with severe pneumonia [31].

The hazard of mortality was 2.43 times higher among patients with a platelet count below < 150,000/µL than the reverse group. Likewise, previous studies also report the same findings [15, 32, 33, 34]. Various mechanisms are involved in the development of thrombocytopenia in individuals with sepsis. During sepsis, platelets are thought to become activated and adhere to the endothelium, resulting in their retention and breakdown. Additionally, immune‐related processes such as the presence of nonspecific antibodies associated with platelets and the cytokine‐driven phagocytosis of platelets can also play a role in thrombocytopenia associated with sepsis [35]. In addition, other literature has also demonstrated the link between low platelet counts and disseminated intravascular coagulation, as well as severe sepsis, thereby leading to poor outcomes [36]. In addition, a low platelet‐to‐lymphocyte ratio was found to be one of the prognostic indicators in patients with severe pneumonia [37]. Clinically, monitoring this ratio could help identify high‐risk patients early, allowing for timely interventions and more targeted treatments, ultimately improving survival rates. Thus, these findings underscore the important of incorporating routine platelet monitoring into the standard care protocol for patients presenting with pneumonia. This proactive approach allows for the early identification of individuals at heightened risk of poor outcomes due to thrombocytopenia.

The multicenter design of the study is a notable strength, as it allows for broader generalizability of the findings beyond individual healthcare facilities. By including multiple centers, the study captures a more diverse patient population, increasing the robustness and applicability of the results to different clinical contexts. One of the main limitations of this study is the potential for unaccounted confounders affecting the observed connection between creatinine levels and mortality. Additionally, the retrospective nature of the study poses certain limitations. Relying on retrospective data hinders the exploration of the effects of causative organisms on biomarkers and, consequently, on pneumonia outcomes.

## 5 Conclusion and Recommendations

This study identified CHF, DM, COPD, thrombocytopenia, and elevated creatinine levels as significant determinants of poor outcomes in older adults with severe pneumonia. Including these factors in the severity assessment is critical to improve risk stratification and patient management. Healthcare providers should routinely evaluate comorbidities such as CHF, DM, and COPD during clinical assessments, as these conditions significantly contribute to poorer prognoses. Simultaneously, regular monitoring of platelet and creatinine levels should be prioritized to identify high‐risk patients promptly. Early identification and management of these risk factors can guide timely and tailored interventions, potentially improving survival rates and overall outcomes in this vulnerable population. Moreover, implementing standardized protocols that incorporate these variables into routine practice could enhance decision‐making and optimize resource allocation in healthcare settings. Further research is warranted to develop specific intervention strategies targeting these determinants and to evaluate their effectiveness in reducing the burden of severe pneumonia‐associated complications and mortality among older adults.

## Author Contributions

**Tegene Atamenta Kitaw:** writing – original draft, conceptualization, investigation, methodology, visualization, writing – review and editing, formal analysis, data curation, software. **Addisu Getie:** writing – original draft, writing – review and editing, project administration, software, methodology. **Melesse Abiye Munie:** writing – original draft, writing – review and editing, methodology, formal analysis. **Yabibal Asfaw Derso:** writing – review and editing, writing – original draft, conceptualization, methodology, formal analysis. **Solomon Gebremichael Asgedom:** writing – original draft, writing – review and editing, resources, formal analysis, methodology, conceptualization. **Ribka Nigatu Haile:** conceptualization, funding acquisition, writing – original draft, methodology, validation, visualization, software, supervision, data curation, writing – review and editing.

## Ethics Statement

The study was reviewed and granted a waiver for the requirement of informed consent by the Woldia University Institutional Review Board (Protocol Number: WDU/IRB001).

## Conflicts of Interest

The authors declare no conflicts of interest.

## Transparency Statement

The lead author Tegene Atamenta Kitaw affirms that this manuscript is an honest, accurate, and transparent account of the study being reported; that no important aspects of the study have been omitted; and that any discrepancies from the study as planned (and, if relevant, registered) have been explained.

## Supporting information

Supplementary_file_1_edited.

Supplementary_file_2_edited.

## Data Availability

Data are available upon reasonable requests.
