# Supplementary material for: Predictors of Poor Pneumonia Outcomes in Older Adults: A Multicentered Follow‐Up Study
Source: Health Sci Rep. 2025 Apr 18;8(4):e70666. doi: 10.1002/hsr2.70666 (PMC12007423; doi:10.1002/hsr2.70666)
Supplement: Supplementary file 1 — Supplementary_file_1_edited. [file HSR2-8-e70666-s002.docx]

***Supplementary file 1: Schoenfeld residuals tests***

**Table 1:Results of assumptions for the Cox proportional hazards model using Schoenfeld residuals tests.**

| Variables | rho | chi2 | df | Prob>chi2 |
| --- | --- | --- | --- | --- |
| Sex | -0.00562 | 0.01 | 1 | 0.9134 |
| Age | -0.05218 | 0.97 | 1 | 0.3248 |
| Chest pain | 0.25885 | 22.80 | 1 | 0.1007 |
| Oxygen saturation | 0.01790 | 0.11 | 1 | 0.7390 |
| Congestive heart failure | 0.02228 | 0.19 | 1 | 0.6602 |
| Diabetes mellites | 0.12091 | 5.39 | 1 | 0.1203 |
| COPD | 0.15167 | 7.97 | 1 | 0.1047 |
| Comorbidity | 0.03000 | 0.36 | 1 | 0.5476 |
| Number of comorbidities | -0.05087 | 0.89 | 1 | 0.3463 |
| White blood cell count | 0.01036 | 0.04 | 1 | 0.8466 |
| Ceftriaxone | -0.02017 | 0.14 | 1 | 0.7084 |
| Vancomycin | -0.19818 | 18.12 | 1 | 0.2091 |
| Global test | | 55.69 | 12 | 0.1379 |
