# Supplementary material for: Predictors of Poor Pneumonia Outcomes in Older Adults: A Multicentered Follow‐Up Study
Source: Health Sci Rep. 2025 Apr 18;8(4):e70666. doi: 10.1002/hsr2.70666 (PMC12007423; doi:10.1002/hsr2.70666)
Supplement: Supplementary file 2 — Supplementary_file_2_edited. [file HSR2-8-e70666-s001.docx]

**Supplementary File 2: Multicollinearity test**

Variance inflation factor and tolerance values were used to check the existence of multicollinearity between variables. A VIF above 4 or tolerance below 0.25 indicated that multicollinearity might exist. In this study, the maximum VIF was 3.33 with a mean VIF of 1.52, and the minimum tolerance value was 0.3. Thus, there is no multicollinearity between covariates.

**Table 1: Multicollinearity test to examine the relationship between explanatory variables.**

| *Variable* | *VIF* | *1/VIF* |
| --- | --- | --- |
| Numbers of comorbidity | 3.33 | 0.300685 |
| Comorbidity | 2.97 | 0.336603 |
| Diabetes mellites | 1.34 | 0.745111 |
| Congestive heart failure | 1.22 | 0.817740 |
| WBC | 1.19 | 0.843182 |
| Creatinine level | 1.18 | 0.849741 |
| Platelet count | 1.14 | 0.880877 |
| Blood urea nitrogen | 1.09 | 0.921608 |
| COPD | 1.08 | 0.922588 |
| Age | 1.08 | 0.926517 |
| Sex | 1.07 | 0.937254 |
| Mean VIF | 1.52 |  |
